# Functional characterization of AarMIXTAs as essential regulators in T-shaped non-glandular trichome development of *Artemisia argyi*

**DOI:** 10.1007/s44307-025-00077-5

**Published:** 2025-09-12

**Authors:** Xinlian Chen, Duan Wu, Chunyu Li, Baosheng Liao, Qi Shen

**Affiliations:** 1 Institute of Medical Plant Physiology and Ecology, School of Pharmaceutical Sciences, Guangzhou University of Chinese Medicine, Guangzhou, 510006 China; 2 Key Laboratory of Quality Evaluation of Chinese Medicine of the Guangdong Provincial Medical Products Administration, the Second Clinical College, Guangzhou University of Chinese Medicine, Guangzhou, 510006 China

**Keywords:** *Artemisia argyi*, T-shaped non-glandular trichomes, *AarMIXTA*s, WGCNA

## Abstract

Trichomes are crucial for plant defense and secondary metabolite biosynthesis. In *Artemisia argyi*, T-shaped non-glandular trichomes (TSTs) are a defining morphological feature and the primary structural component of moxa floss. We observed pronounced TST accumulation on the lower leaf surfaces. To elucidate the genetic regulation of TST development, we performed comparative transcriptomics of TSTs and non-TST tissues. This identified several MIXTA-like transcription factors (named AarMIXTAs) as key regulators of TST differentiation. Phylogenetic analyses revealed gene expansion and functional divergence between the AarMIXTAs and their homologs in *Artemisia annua*. Heterologous overexpression of *AarMIXTA1.2* in *Arabidopsis* significantly increased TST density, demonstrating its positive regulatory role via transcriptional activation of downstream targets. These findings elucidate molecular mechanisms underlying TST morphogenesis and provide a genetic framework for enhancing moxa floss yield in *A. argyi* cultivars.

**Supplementary Information:**

The online version contains supplementary material available at 10.1007/s44307-025-00077-5.

## Introduction

Trichomes are epidermal appendages found on the aerial surfaces of plants (Ishida et al. 2008). They occur widely across most angiosperm, as well as in some gymnosperms and bryophytes, and exhibit diverse morphological forms —including cucumber fruit spines and cotton fibers (Han et al., 2022). These structures are essential for plant environmental adaptation and constitute a primary defense against both biotic and abiotic stresses (Feng et al. 2021). Based on secretory capacity, trichomes are generally classified into glandular secretory trichomes (GSTs) and non-glandular trichomes (NGTs)(Liu et al. 2017). While NGTs lack secretory activity, they provide critical physical defense by reducing transpirational water loss, capturing fog moisture, enhancing extreme temperature tolerance, and shielding against UV radiation and herbivory (Karabourniotis et al. 1992).

*Artemisia argyi* Lévl. et Vant., a perennial herb within the Asteraceae family, is widely recognized in traditional Chinese medicine (TCM) for its therapeutic properties (Sciences et al. 1991). Its dried leaves serve as the primary medicinal material in China, documented to warm the meridians, stop bleeding, dispel cold, relieve pain; and—when applied externally—alleviate dampness and pruritus (Commission C P. 2020). *A. argyi* is extensively utilized in medicine, food, and cultural practices. Its germplasm resources are predominantly distributed in China, Mongolia, and North Korea, and the species is also cultivated in Japan (Sciences et al. 1991). In China, notable regional varieties include “Qi mugwort” from Qichun, Hubei Province, and “Nanyang mugwort” from Nanyang, Henan Province. In 2023, China’s mugwort sector exceed an industrial output value of ¥30 billion. Mugwort is central to treatment and healthcare through moxibustion therapy. This traditional Chinese therapeutic practice, with a history spanning millennia, involves burning *A. argyi* leaf-derived moxa floss to stimulate acupuncture points or warm the body surface for disease prevention and treatment (Chang et al. 2012). The dried leaves are processed into moxa floss, which primarily consists of fibrous tissues—notably leaf trichomes. Moxibustion efficacy is attributed not only to the thermal energy generated during combustion but also to the release of bioactive volatile compounds derived from these trichomes.

*A. argyi* possesses abundant NGTs. Morphologically characterized as T-shaped non-glandular trichomes (TSTs) based on their distinct shape, these trichomes are particularly enriched on the lower leaf surface (Cui et al. 2022). Scanning electron microscopy (SEM) analysis revealed key morphological features: high density on the lower epidermis and elongated structures that frequently intertwine. These TSTs comprise the primary tissue source of moxa floss. The marked asymmetry in TST distribution between the upper and lower leaf surfaces establishes *A. argyi* as an excellent model for studying multicellular TST development. Critically, both the yield and quality of moxa floss—directly determined by trichome abundance and morphology—are critical determinants of moxibustion’s clinical efficacy.

The development of NGTs has primarily been studied in model systems like *Arabidopsis* rosette leaves and cotton fibers, where NGTs are unicellular and originate from single epidermal cells (Wang et al. 2019). This unicellular nature renders them highly tractable for genetic, genomic, and cellular analyses, establishing these species as valuable model systems for elucidating molecular mechanisms of cell differentiation and morphogenesis (Wang et al. 2019). However, no widely adopted model currently exists for studying multicellular NGTs. Trichome development is governed by a complex molecular regulatory network, extensively characterized in *Arabidopsis* and *Artemisia annua *(Yan et al. 2018). A core regulatory module is the MYB-bHLH-WD40 (MBW) transcriptional complex, comprising positive regulators GLABRA1 (GL1), GLABRA3 (GL3)/ENHANCER OF GLABRA3 (EGL3), and TRANSPARENT TESTA GLABRA1 (TTG1). This complex activates downstream transcription factors (TFs) such as GL2 and TTG2 (Yang and Ye 2013). Additionally, R2R3-MYB and HD-ZIP IV TFs play essential roles in initiating and differentiating glandular trichomes in species like *A. annua* and tomato (Chalvin et al. 2020). In 1994, Noda et al. first cloned the *MIXTA* gene from *Antirrhinum majus* that controls flower color intensity and petal surface texture (Noda et al. 1994). *MIXTA* belongs to subgroup 9A (SBG9-A) of the R2R3-MYB family and governs petal epidermal cell differentiation (Brockington et al. 2013). Subsequent studies have identified *MIXTA*/*MIXTA-like* genes as key regulators of epidermal cell patterning across diverse species, where they often directly control trichome development (Lashbrooke et al. 2015; Wu et al. 2018; Zhao et al. 2020).

Despite the biological significance of *A. argyi*, the molecular mechanisms governing trichome formation and regulation remain largely unexplored. In this study, we characterized the epidermal microstructures of *A. argyi* leaves and performed comparative transcriptomic analysis of TST vs non-TST tissues. Key regulatory factors were identified, among which members of the AarMIXTA TF family emerged as central regulators. These candidates were subsequently subjected to evolutionary, expression, and functional analyses. Collectively, this work advances our understanding of trichome development in plants and provides a solid theoretical framework for the metabolic regulation of trichome biosynthesis. These findings offer valuable insights for improving the yield and quality of *A. argyi* and its derived products.

## Materials and methods

### Microscopic observation of *A. argyi* leaves

Two healthy and fully expanded *A. argyi* leaves at the same developmental stage were collected from plants grown in a screenhouse. Trichome distribution on both upper and lower epidermal surfaces was initially assessed using a stereomicroscope. For detailed examination, leaf segments (0.5 cm × 0.5 cm) were excised, mounted on glass slides with distilled water, coverslipped, and observed under a fluorescence microscope. For SEM, two to three leaves were collected (avoiding main veins) and cut into 0.4 cm × 0.4 cm squares. Samples were immediately immersed in 5% glutaraldehyde and fixed at 4℃ for 14 days.

Then fixative was removed, and samples were rinsed three times (10 min per rinse) with 0.1 M phosphate-buffered saline (PBS). Samples were dehydrated through a graded ethanol series (30%, 50%, 70%, and 90%—10 min per concentration) at room temperature, followed by three 10-min rinses in absolute ethanol. Dehydrated samples underwent critical point drying. Dried samples were mounted on stubs, and sputter-coated with gold using an ion sputter coater. Trichome structures, including GSTs and TSTs, were imaged under SEM at various magnifications.

### RNA-seq and transcriptomic analysis

TSTs and non-TST tissues (remaining leaf material after TST removal) from *A. argyi* leaves were carefully separated. Three biological replicates were prepared for each tissue type. Total RNA was extracted using the RNAprep Pure Plant kit (TIANGEN, China) following the manufacturer’s protocol. RNA integrity was verified via agarose gel electrophoresis and spectrophotometry. High-quality total RNA was used as input for library construction. Poly(A) + mRNA was enriched, fragmented, and reverse-transcribed into double-stranded cDNA. Libraries were prepared and sequenced on the Illumina HiSeq X Ten platform in 150 bp paired-end mode. Raw reads were quality-trimmed to remove adapters and low-quality bases. Clean reads were aligned to the *A. argyi* reference genome using HISAT2 (Kim et al. 2019). Transcript assemblies were generated using StringTie (Pertea et al. 2016). Gene-level read counts were obtained using featureCounts. Expression levels were normalized as fragments per kilobase of exon per million mapped reads (FPKM). Subsequent analyses included clustering, quantification of expressed genes, identification and distribution analysis of differentially expressed genes (DEGs), and Gene Ontology (GO) enrichment analysis to compare transcriptomic differences between TSTs and non-TSTs.

### WGCNA of transcriptome

To investigate gene regulatory networks underlying trichome development, publicly available RNA-seq datasets from multiple *A. argyi* tissues were downloaded from National Center for Biotechnology Information (NCBI) (Table S1). These datasets were integrated for expression trend analysis and Weighted Correlation Network Analysis (WGCNA). Co-expression networks were constructed using the WGCNA R package. WGCNA of scale-free to generate modules among genes based on the topological overlap measure (TOM) with default parameters. Transcriptional regulatory networks were built by calculating *Pearson* correlation coefficient (PCC > 0.85) between TFs and other genes within the same modules. In addition, *cis*-regulatory elements in the promoter regions of key genes were predicted to support the inferred regulatory interactions. The final gene-TF regulatory networks were visualized using Cytoscape (Kohl et al., 2010).

### Identification and analysis of homologous genes involved in trichome development

A high-quality genome of autotetraploid *A. argyi* was generated de novo by our research group. To identify trichome development genes and TFs in *A. argyi*, we performed homology-based screening using well-characterized trichome regulators from *Arabidopsis* and *A. annua* as references. Based on known regulatory pathways described in the literature, we reconstructed a putative trichome development pathway for *A. argyi*. Spatial expression patterns of candidate genes were analyzed across multiple tissues. Heatmaps were generated to visualize tissue-specific expression profiles and identify trichome-enriched transcripts. Furthermore, correlation analyses were performed to explore potential associations between TFs and trichome development genes, aiming to identify key regulators within the gene network of *A. argyi*.

### Phylogenetic analysis of MIXTA/MIXTA-like proteins among multispecies

To elucidate evolutionary relationships within the MIXTA/MIXTA-like TF family, we compiled 144 protein sequences from NCBI and Phytozome databases based on published studies (Brockington et al. 2013; Bedon et al. 2014). The dataset comprised 136 angiosperm sequences (21 monocots, 114 dicots, one magnoliid), two gymnosperms, and six ANA-grade species, which were used as outgroups. A maximum likelihood (ML) tree phylogenetic tree was reconstructed using IQ-TREE with default parameters The resulting tree was visualized and annotated in iTOL (https://itol.embl.de/) (Minh et al. 2020).

### Cloning and heterologous transformation of *AarMIXTA1.2*

Total RNA extracted from *A. argyi* was reverse-transcribed into cDNA using the PrimeScript RT Reagent Kit (TaKaRa, China). Gene-specific primers were designed for qRT-PCR of candidate genes (Table S2). Each 10 μL qRT-PCR reaction contained 4.4 μL cDNA template, 0.3 μL forward/reverse primer, and 5 μL 2 × SYBR Green Pro Taq HS Premix (Accurate, China). Gene expression levels were quantified using the 2 − ΔΔCt method. Data presented mean ± standard deviation (SD) of three biological replicates. For functional validation, the full-length *AarMIXTA1.2* coding sequence was cloned sequentially into the intermediate vector Blunt and then into the binary vector pCAMBIA2300, resulting in the recombinant plasmid pCAMBIA2300-Blunt-*AarMIXTA1.2*. The recombinant plasmid was introduced into *Agrobacterium*, which was subsequently used to transform *Arabidopsis *via the floral dip method. Seeds were harvested at maturity and screened for positive transgenic lines. Expression of the transgene in *Arabidopsis* was confirmed by RT-qPCR using the same SYBR Green Pro Taq HS premix qPCR kit according to the instructions. Relative gene expression levels were quantified using the 2–ΔΔCt method. Phenotypic observation of transgenic *Arabidopsis* plants was performed using the same procedures described in the “Microscopic Observation of *A. argyi* Leaves” section.

## Results

### Trichome morphology and distribution in *A. argyi* leaves

**Fig. 1:**
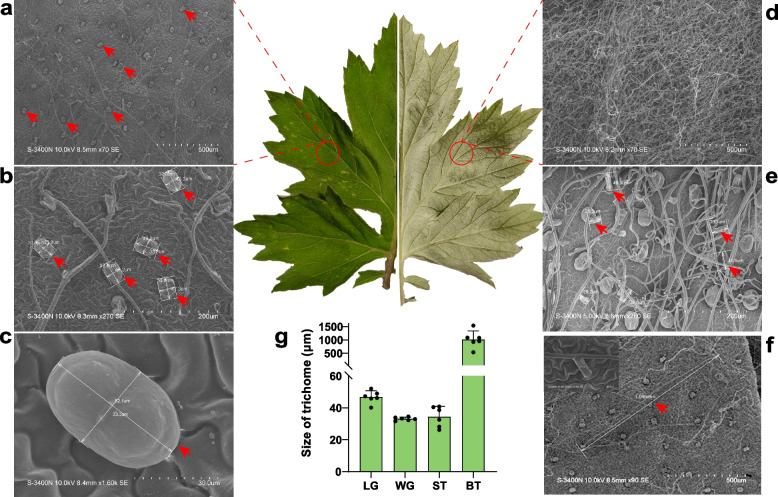
Characteristic of trichomes of *A. argyi* leaves using scanning electron microscope. **a**, **b**, **c** GSTs of upper epidermis of *A. argyi* leaves at different magnifications. **d**, **e** TSTs of lower epidermis of *A. argyi* leaves at different magnifications. **f** The branching length of the TSTs at different magnifications. The upper left corner of the image showed the stalk length of the TST. **g** The size of trichomes on upper surface in *A. argyi* with six biological replicates. LG: length of GSTs; WG: width of GSTs; ST: stalk length of TSTs; BT: branching length of TSTs

Trichomes constitute the primary structural component of moxa floss and represent a defining anatomical feature of *A. argyi*. To characterize their morphology and spatial organization, we examined upper and lower leaf surfaces using fluorescence microscopy and SEM. The upper epidermis exhibited dark green, contrasting with the grayish-green lower surface (Fig. S1). SEM analysis revealed a high density of GSTs on the upper epidermis, while TSTs were comparatively sparse in this region (Fig. 1a). In contrast, the lower epidermis displayed a dense and entangled network of TSTs, with GSTs scattered sporadically. The morphologies of both trichome types were clearly visible under SEM and fluorescence microscopy. GSTs exhibited a slipper-like shape, while TSTs possessed two asymmetric arms.

Detailed SEM observations confirmed the presence of both trichome types on mature *A. argyi* leaves. GSTs measured approximately 33.3 μm × 52.1 μm, while TSTs exhibited stalk lengths of ~ 28.8 μm with branched arms exceeding 1 mm in length (Fig. 1b, c, e, f, g). Trichome density and abundance on the upper surface significantly exceeded those observed in *Arabidopsis* and *A. annua* (Fig. S2). Notably, the lower epidermis displayed exceptionally high density of elongated TSTs that were tightly intertwined, complicating morphological discrimination during quantification (Fig. 1d, e, f).

### Expression differences between TSTs and non-TSTs of *A. argyi* leaves

**Fig. 2:**
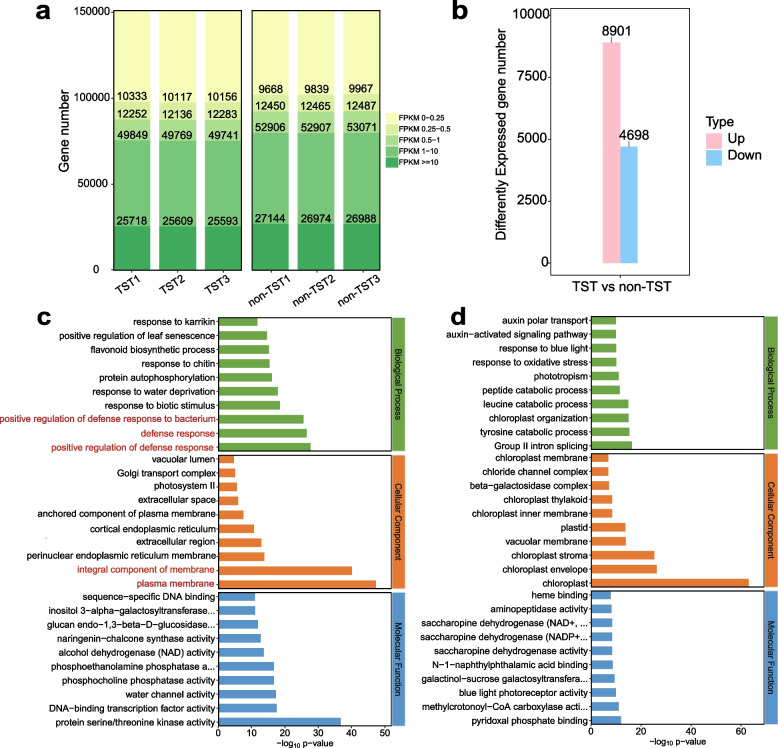
Gene expression of TST and non-TST in *A. argyi*. **a** The FPKM ratio of total genes. **b** The numbers of DEGs between TSTs and non-TSTs. Value parameter: *q*-value < 0.05 and |log2FC|> 1. **c** Top 30 GO term of up-expression genes of TSTs vs non-TSTs. **d** Top 30 GO term of down-expression genes of TSTs vs non-TSTs

To investigate the regulatory mechanisms underlying the high abundance of TSTs in *A. argyi*, TSTs were manually separated from the lower leaf epidermis and subjected to RNA-seq. Gene expression levels were predominantly medium to high (Fig. 2a). Sequencing generated a total of 39.03 Gb of clean reads, with individual samples yielding between 6.27 Gb and 6.88 Gb of high-quality data. Alignment of reads to the *A. argyi* reference genome showed mapping rates ranging from 74.30% to 97.15% (Table S3 and Table S4). The three biological replicates for each tissue type exhibited high consistency, indicating robust data reliability (Fig. S3). Comparative transcriptome analysis identified 8,901 upregulated and 4,698 downregulated genes in TSTs relative to non-TSTs (Fig. 2b). Gene Ontology (GO) enrichment analysis of the upregulated genes revealed significant categories including “integral component of membrane”, “plasma membrane”, and “defense response”, suggesting their relevance to TST development and function (Fig. 2c, d).

Notably, several TF families associated with trichome development were markedly upregulated in TSTs. These included R2R3-MYB proteins, C2H2 zinc-finger proteins, homeodomain-leucine zipper (HD-ZIP) IV proteins, basic helix-loop-helix (bHLH) domain-containing proteins, ethylene-responsive TFs, and WD40 repeat-like superfamily proteins (Yang and Ye 2013; Chalvin et al. 2020). KEGG enrichment analysis of DEGs further indicated significant involvement in pathways such as MAPK signaling, isoflavonoid, and flavonoid biosynthesis. These differences reflect functional divergence between TSTs and non-TST tissues, particularly concerning processes typically associated with glandular trichomes (GSTs), such as secondary metabolite synthesis, storage, and signal transduction.

### Transcription factor and homologous genes analysis

**Fig. 3:**
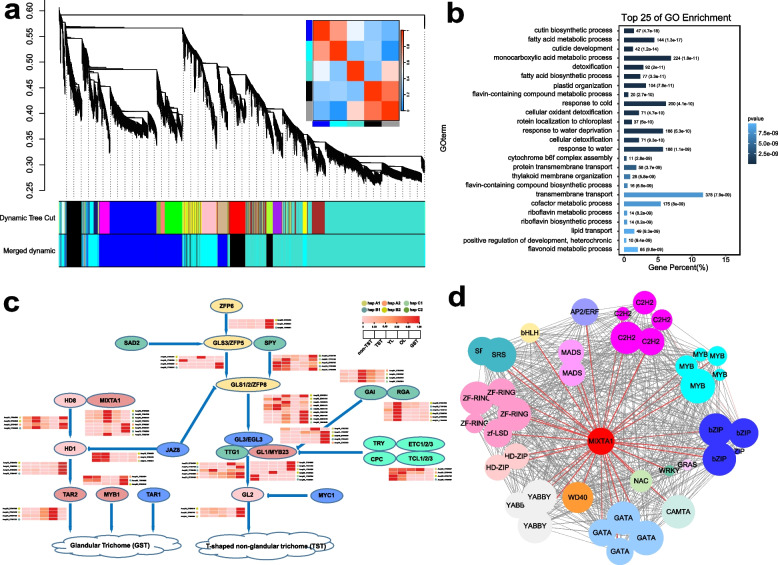
WGCNA of genes in *A. argyi*. **a** Module partitioning, clustering and correlation between modules. **b** GO term of Module cyan. **c** Identification and expression analysis of homologous TFs regulating glandular development in *A. argyi*. **d** WGCNA of trichome regulatory TFs in *A. argyi*

To identify TFs involved in the regulation of trichome development regulation, we integrated transcriptome data from *A. argyi* old leaves (OL), young leaves (YL), stem (St), and GSTs from NCBI databases, alongside our own data from TSTs and non-TSTs (Table S1 and Fig. S4). We performed WGCNA using 53,354 genes, identifying five distinct gene modules (Fig. 3a). The cyan module (MEcyan) was significantly enriched in genes related to the cutin biosynthetic process and contained homologs of key *Arabidopsis* trichome development genes (Fig. 3b). Visualization of the MEcyan co-expression network revealed that *AarMIXTA*s members functioned as hub genes, exhibiting strong associations with multiple other TF families, including MYB, bHLH, WD40, HD-ZIP, and WRKY (Fig. 3d).

Given the well-characterized roles of key TFs in trichome formation in *Arabidopsis* and *A. annua *(Yang and Ye 2013; Yan et al. 2018; Chalvin et al. 2020), we identified 119 *A. argyi* genes associated with trichome development through homologous alignment, representing 27 allelic groups (Table S5). Among these, 14 groups—including *MYB*, *HD-ZIP*, *GIS*, and *ZFP* families—contained four homeologous alleles, while six groups (e.g., *GL1*, *GL2*) possessed three alleles, suggesting gene loss events. Specific gene exhibited distinct duplication patterns: *ETC1*, *TT8*, and *EGL3* underwent tandem duplication, whereas *TTG1*, *RGA*, and *MIXTA*s showed allelic amplification. Tissue-specific expression analysis revealed largely distinct patterns for most trichome-related genes (Fig. 3c). Major homologous gene copies located on homologous chromosomes displayed consistent expression trends (e.g., *ZFP6*, *HD1*, *TAR2*, *JAZ8*, *GAI*, and *RGA*). However, significant expression divergence was observed between homoeologs of several genes, including *MIXTA1*, *HD8*, *GLS1/2*, and *ZFP8* (Fig. 3c).

### Evolutionary relationships of *MIXTA/MIXTA-like* genes

**Fig. 4:**
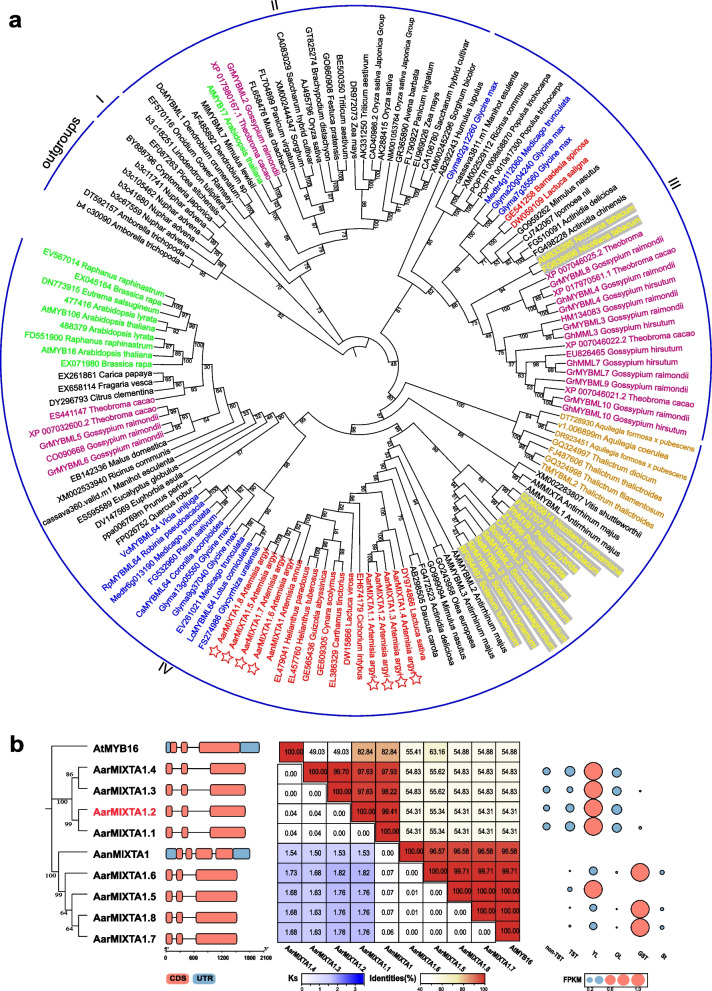
Phylogenetic analysis. **a** The ML tree of MIXTA/MIXTA-like proteins of multispecies. Red, green, blue, purple, orange, and yellow fonts represent species from Asteraceae, Brassicaceae, Fabaceae, Malvaceae, Ranunculaceae, and Solanaceae families. Red stars indicate eight MIXTA proteins of *A. argyi*. **b** Relationship, gene structure, similarity, and differential expression in various tissues of *AarMIXTA* allelic genes

To investigate the evolutionary relationships of *MIXTA*/*MIXTA-like* genes, we constructed an ML phylogenetic tree using 144 MIXTA/MIXTA-like protein sequences obtained from NCBI and Phytozome (Brockington et al. 2013; Bedon et al. 2014) (Fig. 4a and Table S6). The dataset comprised sequences from 136 angiosperms, two gymnosperms, and six ANA-grade species as outgroups. The angiosperm sequences included 21 monocots, 114 dicots, and one magnoliid. Excluding the outgroups, the sequences clustered into four major clades (I-IV) (Fig. 4a). Clade I contained only the two gymnosperm proteins, representing the most ancestral lineage. Clade II comprised 21 monocots, four dicots, and one magnoliid species, also represents an early divergence. Clades III and IV, containing 33 and 77 proteins respectively, consisted exclusively of dicots. This suggests that Clades III and IV likely originated from Clades I and II, with proteins in Clades I and II predating the monocot-dicot divergence. Species-specific clustering patterns were evident, with many lineages showing evidence of ancestral *MIXTA/MIXTA-like* gene acquisition followed by lineage-specific duplication or expansion. Clear intra-family clustering was observed within Asteraceae, Brassicaceae, Fabaceae, Malvaceae, Ranunculaceae, and Solanaceae. The overall phylogenetic structure of MIXTA/MIXTA-like proteins across families was consistent with the APG IV classification system (https://www.mobot.org/MOBOT/research/APweb/). As MIXTA/MIXTA-like proteins belong to SBG9 family of TFs, the evolutionary relationships of these 144 family members also reflect key evolutionary characteristics of the SBG9 subfamily to a certain extent.

Homology-based analysis identified eight *AarMIXTA* genes within the *A. argyi* genome, indicating a substantial expansion compared to *A. annua*. Phylogenetic analysis grouped these genes clustered into two distinct clades. The first clade (*AarMIXTA1.1–1.4*) exhibited closest affinity to *Lactuca sativa*, while the second clade (*AarMIXTA1.5–1.8*) showed higher similarity to *A. annua*. This clustering pattern suggests that the duplication events leading to the *AarMIXTA* expansion likely occurred after the divergence of the Asteraceae lineage from other eudicots (Fig. 4a). Analysis of synonymous substitution rates (*Ks*) between the two clades revealed a relatively high degree of nucleotide divergence (Fig. 4b), indicating accelerated evolution within these gene regions. This acceleration is potentially attributable to localized elevated recombination rates, relaxed selective constraints, or positive selection. Furthermore, the elevated *Ks* values support the contribution of segmental or tandem duplication events to the expansion of the *MIXTA-like* gene family in *A. argyi*.

Although the *AarMIXTA* genes share conserved exon–intron structures and high sequence similarity with their *AanMIXTA* homologs (Fig. 4b and Fig. S5), they exhibit markedly distinct expression profiles (Fig. 4b). Specifically, the clade comprising *AarMIXTA1.1* to *1.4* showed significantly higher expression in TSTs, while *AarMIXTA1.5* to *1.8* were more abundantly expressed in GSTs (Fig. 4b). *AanMIXTA1* in *A. annua* is a functionally validated positive regulator of GST density. Given the close phylogenetic relationship, its homologous cluster—*AarMIXTA1.5* to *1.8*—likely share this regulatory role in GST development. In contrast, the other clade (*AarMIXTA1.1* to *1.4*) occupies a distinct phylogenetic position, suggesting it may regulate different biological processes, potentially specific to TST development. These findings indicate functional divergence following gene duplication within the *MIXTA/MIXTA-like* family, with the two AarMIXTA clades potentially specializing in regulating distinct trichome types (TSTs vs. GSTs) in *A. argyi*.

### In vivo functional assay of *AarMIXTA1.2* in transgenic *Arabidopsis*

**Fig. 5:**
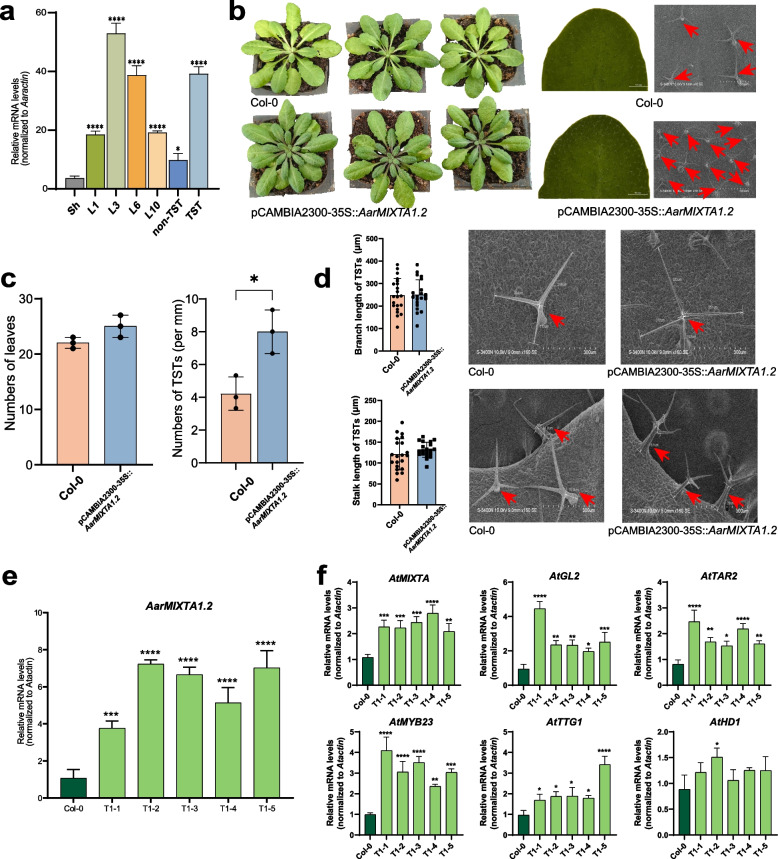
The transgenic *Arabidopsis* overexpressing *AarMIXTA1.2*. **a** Expression trend of *AarMIXTA1.2* in different leaf developments, TSTs and non-TSTs. Sh, L1, L3, L6, L10 represented leaf buds, the first group, the third group, the sixth group, the tenth group of leaves, respectively. **b** The seeding, leaves and its upper epidermis with SEM of Col-0 and over-expression pCAMBIA2300-35S::*AarMIXTA1.2 Arabidopsis*. **c** Changes in leaves numbers and TSTs density in transgenic *Arabidopsis* leaves. **d** The change of branch and stalk length in transgenic *Arabidopsis* leaves. The red arrows in b and d point to TSTs. **e** Gene expression of over-expression pCAMBIA2300-35S::*AarMIXTA1.2 Arabidopsis* using qRT-PCR. **f** The expression leaves of key TFs responding to *AarMIXTA1.2* in *Arabidopsis* using qRT-PCR

To investigate the functional role of the *AarMIXTA1.1–1.4* clade, *AarMIXTA1.2* was selected as a representative gene based on its expression profile. qRT-PCR analysis revealed progressive upregulation of *AarMIXTA1.2* during leaf development, peaking in the third group of leaves. Moreover, *AarMIXTA1.2* exhibited strong and specific expression in TSTs versus non-TST tissues, confirming its importance in TST development (Fig. 5a and Table S6). The gene was subsequently cloned and transformed into *Arabidopsis* (Fig. 5b and Fig. S6).

qRT-PCR confirmed significantly elevated *AarMIXTA1.2* transcript levels in transgenic lines (pCAMBIA2300-35S::*AarMIXTA1.2*) compared to wild-type Col-0, verifying successful transgene integration and expression (Fig. 5e). Phenotypic assessment revealed two key alterations in transgenic plants: Increased total leaf number and significantly enhanced trichome density on leaves (Fig. 5c). Stereomicroscopy and SEM analysis coupled with quantitative morphometry demonstrated that TST density in transgenic plants approximately doubled relative to wild-type controls. Branch and stalk lengths of trichomes were also moderately increased (Fig. 5d).

To elucidate the regulatory mechanism, expression of core Arabidopsis trichome development genes (*AtGL2*, *AtTAR2*, *AtMYB23*, *AtTTG1*, and *AtHD1*) were analyzed. All genes showed significantly upregulated expression in *AarMIXTA1.2*-overexpressing plants (Fig. 5f). Notably, expression of native *AtMIXTA* gene was also elevated, suggesting potential feedback regulation or synergistic interaction. These results demonstrate that *AarMIXTA1.2* overexpression promote TST development in *Arabidopsis*, likely through activation of a cascade involving key downstream TFs and regulatory genes. This underscores the central role of *AarMIXTA1.2* in modulating trichome development.

## Discussion

The striking polarity in GST and TST distribution between the upper and lower leaf surfaces of *A. argyi* establishes it an ideal model for investigating the development of multicellular TSTs. While regulatory mechanisms for unicellular trichomes are well-characterized in model systems like *Arabidopsis* and cotton, the control of dense, multicellular TSTs exhibiting epidermal polarity remains poorly understood and likely involves more complex regulatory networks. Our study provides a hierarchical approach: detailed morphological characterization, comparative transcriptomics (TSTs vs non-TSTs), phylogenetic analysis of *MIXTA/MIXTA-like* genes, and functional validation of *AarMIXTA1.2*. Collectively, these integrated analyses elucidate key intrinsic factors driving TST development in *A. argyi*.

*MIXTA* genes, belonging to the SBG9 subgroup of R2R3-MYB TFs, are pivotal regulators of epidermal cell differentiation (Brockington et al. 2013). Their role in trichome development is conserved across species with specialized trichomes, such as *AanMIXTA1* promoting GST initiation and artemisinin biosynthesis in *A. annua *(Shi et al. 2018) and *CsMIXTA* regulating glandular trichome morphogenesis in *Cannabis sativa *(Haiden et al. 2022). The defining trait of *A. argyi*—a dense array of elongated TSTs on the lower epidermis—correlates with our discovery of significant expansion and functional diversification within the *AarMIXTA* gene family. This expansion, likely driven by polyploidy events, facilitated gene dosage effects and neofunctionalization. Functional validation demonstrated that *AarMIXTA1.2* overexpression in *Arabidopsis* significantly increased TST density, providing direct evidence for functional divergence among *AarMIXTA* paralogs. Genome-wide analyses further revealed widespread allelic amplification and tandem duplication among trichome-related TFs. Notably, *AarMIXTA*s exhibited balanced allelic amplification coupled with expression divergence among paralogs, positioning them as core regulators orchestrating TST development in *A. argyi*.

This study also opens several promising research avenues. Given *A. argyi*’s remarkable environmental adaptability across diverse Chinese habitats, investigating the potential contribution of trichome development to abiotic stress resilience is warranted. Deeper mechanistic insights into *AarMIXTA* regulation could be gained through studies on protein–protein interactions (e.g., yeast two-hybrid screening to identify AarMIXTA1.2 interactors, followed by validation via co-immunoprecipitation or bimolecular fluorescence complementation), epigenetic modifications, and chromatin accessibility (e.g., ATAC-seq). Functional characterization of interacting partners via overexpression and silencing in *A. argyi* itself will be crucial.

In conclusion, we demonstrate that *AarMIXTA1.2* plays a pivotal role in promoting TST development. Evolutionary analysis reveals a lineage-specific expansion of *MIXTA/MIXTA-like* genes in *A. argyi*, likely underpinning its unique trichome phenotype. These findings significantly advance our understanding of the molecular mechanisms controlling multicellular trichome development and provide a robust mechanistic framework for enhancing *A. argyi* quality and productivity through targeted genetic screening, trait selection, and molecular breeding strategies.

## Supplementary Information

Supplementary Material 1.

Supplementary Material 2. FigS. 1 Microscopic observation of the epidermis of *A. argyi*. a Upper epidermis. b upper epidermis (Bright, fluorescence microscope ×40). c Lower epidermis. d Lower epidermis (Bright, fluorescence microscope ×40).

Supplementary Material 3. FigS. 2 The density of TSTs on upper epidermis (a) and characteristics in (b) *Arabidopsis*, (c) *A. annua *and (d) *A. argyi*.

Supplementary Material 4. FigS. 3 The correlation of sequenced samples.

Supplementary Material 5. FigS. 4 Sample clustering. GST: Trichome (NCBI); St: Stem; non-TST: mesophyll; TST: Trichome (ours); YL: Young_leave; OL: Old_leave.

Supplementary Material 6. FigS. 5 Sequence similarity comparison of MIXTAs in *Arabidopsis*, *A. annua *and* A. argyi*.

Supplementary Material 7. FigS. 6 PCR verification of transgenic 35S::*AarMIXTA1.2Arabidopsis*.

## Data Availability

Table S1. Transcriptome accession number for other tissues in *A. argyi* Table S2. The sequences of primers Table S3. Statistical results of sample transcriptome sequencing Table S4. Statistical results of transcriptomic reads mapping genome Table S5. Identification of genes related to trichome development in *A. argyi* Table S6. Species information of phylogenetic tree
