# Supplementary material for: Functional characterization of AarMIXTAs as essential regulators in T-shaped non-glandular trichome development of Artemisia argyi
Source: Adv Biotechnol (Singap). 2025 Sep 12;3(3):26. doi: 10.1007/s44307-025-00077-5 (PMC12425882; doi:10.1007/s44307-025-00077-5)
Supplement: Supplementary file 1 — Supplementary Material 1. [file 44307_2025_77_MOESM1_ESM.docx]

**Table S1. Transcriptome accession number for other tissues in *A. argyi***

| **Number** | **SRA** | **Tissue** | **Sample** |
| --- | --- | --- | --- |
| 1 | SRX19698237 | Old leave | OL1 |
| 2 | SRX19698234 | Old leave | OL2 |
| 3 | SRX19698228 | Old leave | OL3 |
| 4 | SRX19698236 | Young leave | YL1 |
| 5 | SRX19698235 | Young leave | YL2 |
| 6 | SRX19698233 | Young leave | YL3 |
| 7 | SRX19698232 | Trichome | GST1 |
| 8 | SRX19698231 | Trichome | GST2 |
| 9 | SRX19698230 | Trichome | GST3 |

**Table S2. The sequences of primers**

| gene clone | AarMIXTA1.2-1F | ATGGGAAGATCGCCATGTT |
| --- | --- | --- |
|  | AarMIXTA1.2-1R | CTAAAGCAGAAGAGAACTAGATTG |
| vecter constructure | AarMIXTA1.2-1-KpnI-F | GGGGTACCATGGGAAGATCGCCATGTT |
|  | AarMIXTA1.2-1-BamHI-R | CGGGATCCCTAAAGCAGAAGAGAACTAGATTG |
| qRT-PCR in A. argyi | AarMIXTA1.2-F | AAAGCAAATCATTAGGGTCT |
|  | AarMIXTA1.2-R | GAAGTTATCATTCGGCACAT |
|  | AarActin-F | GTTGCCCAGAGGTCTTGTT |
|  | AarActin-R | TGCTGGAAGGTGCTAAGTG |
| qRT-PCR in Arabidopsis | ArTAR2-F | CATACCCGTTGATTACAGAC |
|  | ArTAR2-R | TGTTTCAATAGTTTCCACCT |
|  | ArGL2-F | TTCGCTGTGGTTACCTGTTT |
|  | ArGL2-R | CATACGAGTTAGTGCTGCTGTC |
|  | ArTTG1-F | AATCCGACTGACACTGACCT |
|  | ArTTG1-R | GCTGTTGTTGAGAACCGAGA |
|  | ArMYB23-F | ACCGACTCAGCCACTTTCTC |
|  | ArMYB23-R | TTTCGCTATCAACGACCATC |
|  | ArHD1-F | GTTGGTCCTAATAATAATCTGC |
|  | ArHD1-R | AAAGTTCTGGTTCTTCCCTC |
|  | ArMIXTA-F | TTCCACAACTGTAGCCAAAC |
|  | ArMIXTA-R | ATGAGTAGCAATCGCTGACC |
|  | ArActin-F | GGTAACATTGTGCTCAGTGGTGG |
|  | ArActin-R | GGTGCAACGACCTTAATCTTCAT |

**Table S3. Statistical results of sample transcriptome sequencing**

| **Sample** | **RawReads** | **RawBases** | **CleanReads** | **CleanBases** | **ValidBases** | **Q30** | **GC** |
| --- | --- | --- | --- | --- | --- | --- | --- |
| non-TST1 | 48.19 Mb | 7.23 Gb | 47.26 Mb | 6.88 Gb | 95.18% | 94.95% | 43.21% |
| non-TST2 | 43.91 Mb | 6.59 Gb | 43.10 Mb | 6.27 Gb | 95.25% | 94.90% | 43.25% |
| non-TST3 | 46.01 Mb | 6.90 Gb | 45.12 Mb | 6.57 Gb | 95.25% | 94.90% | 43.28% |
| TST1 | 44.36 Mb | 6.65 Gb | 43.70 Mb | 6.37 Gb | 95.67% | 94.97% | 43.76% |
| TST2 | 44.35 Mb | 6.65 Gb | 43.67 Mb | 6.34 Gb | 95.38% | 94.99% | 43.76% |
| TST3 | 46.14 Mb | 6.92 Gb | 45.46 Mb | 6.60 Gb | 95.38% | 95.04% | 43.73% |

**Table S4. Statistical results of transcriptomic reads mapping genome**

| **Sample** | **Total reads** | **Total mapped reads (%)** | **Multiple mapped (%)** | **Uniquely mapped (%)** | **Read-1 (%)** | **Read-2 (%)** | **Reads map to '+' (%)** | **Reads map to '-' (%)** | **Non-splice reads (%)** | **Splice reads (%)** | **Reads mapped in proper pairs (%)** |
| --- | --- | --- | --- | --- | --- | --- | --- | --- | --- | --- | --- |
| non-TST1 | 47,256,246 | 97.07 | 39.00 | 58.07 | 29.04 | 29.03 | 29.03 | 29.04 | 36.05 | 22..03 | 57.06 |
| non-TST2 | 43,102,534 | 96.99 | 38.84 | 58.14 | 29.08 | 29.06 | 29.07 | 29.08 | 36.01 | 22.14 | 57.13 |
| non-TST3 | 45,124,022 | 97.15 | 39.16 | 57.99 | 29.00 | 28.98 | 28.99 | 29.00 | 35.88 | 22.11 | 56.98 |
| TST1 | 43,695,160 | 74.30 | 28.89 | 45.41 | 22.71 | 22.70 | 22.70 | 22.71 | 29.74 | 15.67 | 44.62 |
| TST2 | 43,672,124 | 74.46 | 29.68 | 44.79 | 22.40 | 22.39 | 22.39 | 22.40 | 29.45 | 15.34 | 43.93 |
| TST3 | 45,461,870 | 74.57 | 29.67 | 44.90 | 22.46 | 22.45 | 22.45 | 22.46 | 29.58 | 15.33 | 44.04 |

**Table S5. Identification of genes related to trichome development in *A. argyi***

| **Name** | **Family** | ***Arabidopsis*** | ***Artemisia annua*** | ***Artemisia_argyi*** |
| --- | --- | --- | --- | --- |
| SYP | SPINDLY | AT3G11540 | chr4g00735901 | Aarg1B_2T003735.1 |
|  |  |  |  | Aarg1B_1T003669.1 |
|  |  |  |  | Aarg1A_1T003835.1 |
| JAZ1 | Jasmonate ZIM-domain | AT1G19180 | chr4g00725711 | Aarg1B_1T004048.1 |
|  |  |  |  | Aarg1A_1T004234.1 |
|  |  |  |  | Aarg1B_2T004130.1 |
|  |  |  |  | Aarg1A_2T004525.1 |
| JAZ8 | Jasmonate ZIM-domain | AT1G30135 | chr4g00689071 | Aarg1B_2T006225.1 |
|  |  |  |  | Aarg1A_2T006328.1 |
|  |  |  |  | Aarg1A_1T006213.1 |
| TAR2 | Myb Transcription Factor | AT4G24670 | chr1g00024361 | Aarg2B_2T005045.1 |
|  |  |  |  | Aarg2B_1T005648.1 |
|  |  |  |  | Aarg2A_2T004842.1 |
|  |  |  |  | Aarg2A_1T004578.1 |
| RGA | DELLA proteins | AT1G19530 | chr1g00109051 | Aarg3B_1T001636.1 |
|  |  |  |  | Aarg7B_1T005123.1 |
|  |  |  |  | Aarg7B_2T005759.1 |
|  |  |  |  | Aarg7A_2T004656.1 |
|  |  |  |  | Aarg7A_1T004389.1 |
|  |  |  |  | Aarg3B_2T001339.1 |
|  |  |  |  | Aarg3A_1T001726.1 |
|  |  |  |  | Aarg2A_1T000072.1 |
|  |  |  |  | Aarg2B_1T000179.1 |
| EGL3 | basic helix-loop-helix | AT1G63650 | chr5g00866651 | Aarg4A_1T007505.1 |
|  |  |  |  | Aarg4A_1T007507.1 |
|  |  |  |  | Aarg4B_1T006481.1 |
|  |  |  |  | Aarg4B_2T006835.1 |
|  |  |  |  | Aarg4A_2T007360.1 |
| TT8 | basic helix-loop-helix | AT4G09820 | chr2g00411561 | Aarg8C_2T001984.1 |
|  |  |  |  | Aarg8C_1T002341.1 |
|  |  |  |  | Aarg8C_1T002340.1 |
|  |  |  |  | Aarg8B_2T002580.1 |
|  |  |  |  | Aarg8B_1T002096.1 |
| GAI | DELLA proteins | AT1G14920 | chr7g01176981 | Aarg8A_2T002469.1 |
|  |  |  |  | Aarg8C_2T007019.1 |
|  |  |  |  | Aarg8C_1T007504.1 |
|  |  |  |  | Aarg8A_1T002327.1 |
| SAP1 | Secreted aspartic protease 1 | AT1G03230 | chr2g00241511 | Aarg5B_1T005042.1 |
|  |  |  |  | Aarg5A_1T004340.1 |
|  |  |  |  | Aarg5A_2T003777.1 |
| TTG1 | WD40 Transcription Factor | AT5G24520 | chr3g00591531 | Aarg8C_1T008710.1 |
|  |  |  |  | Aarg8B_2T005277.1 |
|  |  |  |  | Aarg8B_1T004415.1 |
|  |  |  |  | Aarg8C_2T008122.1 |
|  |  |  | chr6g01085471 | Aarg2B_2T003997.1 |
|  |  |  |  | Aarg2B_1T004668.1 |
|  |  |  |  | Aarg2A_2T003933.1 |
|  |  |  |  | Aarg2A_1T003580.1 |
| ORA | AP2/ERF transcription factor | - | AGB07586.1 | Aarg5B_1T005318.1 |
|  |  |  |  | Aarg5A_1T004616.1 |
|  |  |  |  | Aarg5B_2T004410.1 |
|  |  |  |  | Aarg5A_2T004056.1 |
| ZFP5 | C2H2 zinc finger protein | AT1G10480 | chr3g00521291 | Aarg4B_2T004932.1 |
|  |  |  |  | Aarg4A_2T005470.1 |
|  |  |  |  | Aarg4B_1T004555.1 |
|  |  |  |  | Aarg4A_1T005521.1 |
| ZFP6 | C2H2 zinc finger protein | AT1G67030 | chr2g00288871 | Aarg5B_2T005580.1 |
|  |  |  |  | Aarg5A_2T005221.1 |
|  |  |  |  | Aarg5B_1T006551.1 |
| GIS | C2H2 zinc finger protein | AT5G06650 | chr1g00049481 | Aarg2A_1T005458.1 |
|  |  |  |  | Aarg2A_2T005734.1 |
|  |  |  |  | Aarg2B_2T005914.1 |
|  |  |  |  | Aarg2B_1T006619.1 |
| GIS |  | AT3G58070 | chr6g01000421 | Aarg6B_2T001431.1 |
|  |  |  |  | Aarg6A_1T002073.1 |
|  |  |  |  | Aarg6A_2T001474.1 |
|  |  |  |  | Aarg6B_1T001734.1 |
| ZFP8 |  | AT2G41940 | p0ctg_886g01510631 | Aarg8A_1T001759.1 |
|  |  |  |  | Aarg8A_2T001942.1 |
|  |  |  |  | Aarg8C_2T006445.1 |
|  |  |  |  | Aarg8C_1T006829.1 |
| NTL8 | NAC transcription factor | AT2G27300 | chr6g01043531 | Aarg2A_2T002613.1 |
|  |  |  |  | Aarg2A_1T002490.1 |
|  |  |  |  | Aarg2B_2T002694.1 |
|  |  |  |  | Aarg2B_1T003394.1 |
| GL2 （GLABRA2） | HD-ZIP IV family, homeodomain protein，class IV homeodomain-Leucine Zipper gene family | AT1G79840 | p0ctg_43g01514741 | Aarg3B_2T000519.1 |
|  |  |  |  | Aarg3A_2T000703.1 |
|  |  |  |  | Aarg3A_1T000598.1 |
| HD8 | HD-ZIP IV family, homeodomain protein，class IV homeodomain-Leucine Zipper gene family | - | chr2g00262181 | Aarg5A_1T004854.1 |
|  |  |  |  | Aarg5B_2T004629.1 |
|  |  |  |  | Aarg5A_2T004257.1 |
|  |  |  |  | Aarg5B_1T005562.1 |
| HD1 | HD-ZIP IV Transcription Factor | AT4G38130 | chr5g00872511 | Aarg4B_2T006433.1 |
|  |  |  |  | Aarg4A_1T007035.1 |
|  |  |  |  | Aarg4A_2T006952.1 |
|  |  |  |  | Aarg4B_1T006107.1 |
| HDG11/12 | HD-ZIP IV family, homeodomain protein，class IV homeodomain-Leucine Zipper gene family | AT1G73360/AT1G17920 | chr3g00541071 | Aarg4A_1T003244.1 |
|  |  |  |  | Aarg4A_2T003414.1 |
|  |  |  |  | Aarg4B_2T002913.1 |
|  |  |  |  | Aarg4B_1T002670.1 |
| ETC1 /TCL2 | R3-MYB Transcription Factor | AT1G01380/AT2G30424 | chr2g00265651/chr2g00265611 | Aarg5B_2T004765.1 |
|  |  |  |  | Aarg5B_1T005687.1 |
|  |  |  |  | Aarg5A_2T004402.1 |
|  |  |  |  | Aarg5A_2T004400.1 |
|  |  |  |  | Aarg5A_1T005007.1 |
|  |  |  |  | Aarg5A_1T005006.1 |
| MYB23 | R2R3-MYB Transcription Factor | AT5G40330 | chr2g00328411 | Aarg8C_2T010177.1 |
|  |  |  |  | Aarg8C_1T011065.1 |
|  |  |  |  | Aarg8A_2T004098.1 |
|  |  |  |  | Aarg8A_1T004066.1 |
| GL1 |  | AT3G27920 | chr7g01240871 | Aarg8B_2T000024.1 |
|  |  |  |  | Aarg8C_1T007524.1 |
|  |  |  |  | Aarg8B_1T000031.1 |
| MYB1 | Myb Transcription Factor | AT3G09230 | chr2g00415671 | Aarg8B_1T001874.1 |
|  |  |  |  | Aarg8B_2T002356.1 |
|  |  |  |  | Aarg8C_1T002137.1 |
|  |  |  |  | Aarg8C_2T001773.1 |
| MYB5 | Myb Transcription Factor | AT3G13540.1 | chr6g01072861 | Aarg2B_2T003625.1 |
|  |  |  |  | Aarg2B_1T004286.1 |
|  |  |  |  | Aarg2A_2T003592.1 |
|  |  |  |  | Aarg2A_1T003194.1 |
| MIXTA1 | R2R3-MYB Transcription Factor | AT5G15310 | chr3g00498371 | Aarg8B_2T005626.1 |
|  |  |  |  | Aarg8B_1T004748.1 |
|  |  |  |  | Aarg8C_2T008444.1 |
|  |  |  |  | Aarg8C_1T009034.1 |
|  |  |  |  | Aarg7A_2T002287.1 |
|  |  |  |  | Aarg7B_2T003401.1 |
|  |  |  |  | Aarg7B_1T002596.1 |
|  |  |  |  | Aarg7A_1T002199.1 |

**Table S6. Species information of phylogenetic tree**

| **Name** | **Family** | **Clade** | **subClade** | **Color in tree** |
| --- | --- | --- | --- | --- |
| b4_c30090_Amborella_trichopoda | Amborellaceae |  | ANA | black |
| DT592157_Amborella_trichopoda | Amborellaceae |  | ANA | black |
| b3c105462_Nuphar_advena | Nymphaeaceae |  | ANA | black |
| b3c11741_Nuphar_advena | Nymphaeaceae |  | ANA | black |
| b3c41680_Nuphar_advena | Nymphaeaceae |  | ANA | black |
| b3c67559_Nuphar_advena | Nymphaeaceae |  | ANA | black |
| FL658476_Musa_chachaco | Musaceae | Angiosperms | Monocots | black |
| AF485892_Dendrobium_sp | Orchidaceae | Angiosperms | Monocots | black |
| DcMYBML1_Dendrobium_crumenatum | Orchidaceae | Angiosperms | Monocots | black |
| EF570116_Oncidium_Gower_Ramsey | Orchidaceae | Angiosperms | Monocots | black |
| AJ495796_Oryza_sativa | Poaceae | Angiosperms | Monocots | black |
| AK288415_Oryza_sativa | Poaceae | Angiosperms | Monocots | black |
| AK331250_Triticum_aestivum | Poaceae | Angiosperms | Monocots | black |
| BE500350_Triticum_aestivum | Poaceae | Angiosperms | Monocots | black |
| CA083029_Saccharum_hybrid_cultivar | Poaceae | Angiosperms | Monocots | black |
| CA106780_Saccharum_hybrid_cultivar | Poaceae | Angiosperms | Monocots | black |
| CAD40986.2_Oryza_sativa_Japonica_Group | Poaceae | Angiosperms | Monocots | black |
| DR972073_Zea_mays | Poaceae | Angiosperms | Monocots | black |
| EU969026_Zea_mays | Poaceae | Angiosperms | Monocots | black |
| FL704899_Panicum_virgatum | Poaceae | Angiosperms | Monocots | black |
| FL790922_Panicum_virgatum | Poaceae | Angiosperms | Monocots | black |
| GO860908_Festuca_pratensis | Poaceae | Angiosperms | Monocots | black |
| GR365890_Avena_barbata | Poaceae | Angiosperms | Monocots | black |
| GT825274_Brachypodium_distachyon | Poaceae | Angiosperms | Monocots | black |
| NM001053764_Oryza_sativa_Japonica_Group | Poaceae | Angiosperms | Monocots | black |
| XM002444347_Sorghum bicolor | Poaceae | Angiosperms | Monocots | black |
| XM002452296_Sorghum_bicolor | Poaceae | Angiosperms | Monocots | black |
| b3_c18251_Liriodendron_tulipifera | Magnoliaceae | Angiosperms | magnoliid | black |
| AMMIXTA_Antirrhinum_majus | Plantaginaceae | Angiosperms | dicots | black |
| AMMYBML1_Antirrhinum_majus | Plantaginaceae | Angiosperms | dicots | black |
| AMMYBML2_Antirrhinum_majus | Plantaginaceae | Angiosperms | dicots | black |
| AMMYBML3_Antirrhinum_majus | Plantaginaceae | Angiosperms | dicots | black |
| FG472523_Actinidia_deliciosa | Actinidiaceae | Angiosperms | dicots | black |
| FG498228_Actinidia_chinensis | Actinidiaceae | Angiosperms | dicots | black |
| FG510091_Actinidia_deliciosa | Actinidiaceae | Angiosperms | dicots | black |
| AB298505_Daucus_carota | Apiaceae | Angiosperms | dicots | black |
| AarMIXTA1.1_Artemisia_argyi | Asteraceae | Angiosperms | dicots | red |
| AarMIXTA1.2_Artemisia_argyi | Asteraceae | Angiosperms | dicots | red |
| AarMIXTA1.3_Artemisia_argyi | Asteraceae | Angiosperms | dicots | red |
| AarMIXTA1.4_Artemisia_argyi | Asteraceae | Angiosperms | dicots | red |
| AarMIXTA1.5_Artemisia_argyi | Asteraceae | Angiosperms | dicots | red |
| AarMIXTA1.6_Artemisia_argyi | Asteraceae | Angiosperms | dicots | red |
| AarMIXTA1.7_Artemisia_argyi | Asteraceae | Angiosperms | dicots | red |
| AarMIXTA1.8_Artemisia_argyi | Asteraceae | Angiosperms | dicots | red |
| AanMIXTA1_Artemisia_annua | Asteraceae | Angiosperms | dicots | red |
| DW059109_Lactuca_saligna | Asteraceae | Angiosperms | dicots | red |
| DW15866_Lactuca_virosa | Asteraceae | Angiosperms | dicots | red |
| DY974866_Lactuca_sativa | Asteraceae | Angiosperms | dicots | red |
| EH674179_Cichorium_intybus | Asteraceae | Angiosperms | dicots | red |
| EL386329_Carthamus_tinctorius | Asteraceae | Angiosperms | dicots | red |
| EL457760_Helianthus_tuberosus | Asteraceae | Angiosperms | dicots | red |
| EL479041_Helianthus_paradoxus | Asteraceae | Angiosperms | dicots | red |
| GE541258_Barnadesia_spinosa | Asteraceae | Angiosperms | dicots | red |
| GE565436_Guizotia_abyssinica | Asteraceae | Angiosperms | dicots | red |
| GE609305_Cynara_scolymus | Asteraceae | Angiosperms | dicots | red |
| 477416_Arabidopsis_lyrata | Brassicaceae | Angiosperms | dicots | green |
| 488379_Arabidopsis_lyrata | Brassicaceae | Angiosperms | dicots | green |
| AtMYB106_Arabidopsis_thaliana | Brassicaceae | Angiosperms | dicots | green |
| AtMYB16_Arabidopsis_thaliana | Brassicaceae | Angiosperms | dicots | green |
| AtMYB17_Arabidopsis_thaliana | Brassicaceae | Angiosperms | dicots | green |
| DN773915_Eutrema_salsugineum | Brassicaceae | Angiosperms | dicots | green |
| EV567014_Raphanus_raphinastrum | Brassicaceae | Angiosperms | dicots | green |
| EX045164_Brassica_rapa | Brassicaceae | Angiosperms | dicots | green |
| EX071980_Brassica_rapa | Brassicaceae | Angiosperms | dicots | green |
| FD551900_Raphanus_raphinastrum | Brassicaceae | Angiosperms | dicots | green |
| AB292243_Humulus_lupulus | Cannabaceae | Angiosperms | dicots | black |
| EX261861_Carica_papaya | Caricaceae | Angiosperms | dicots | black |
| CJ742067_Ipomoea_nil | Convolvulaceae | Angiosperms | dicots | black |
| cassava360.valid.m1_Manihot_esculenta | Euphorbiaceae | Angiosperms | dicots | black |
| cassava3811.m1_Manihot_esulenta | Euphorbiaceae | Angiosperms | dicots | black |
| DV147569_Euphorbia_esula | Euphorbiaceae | Angiosperms | dicots | black |
| XM002529112_Ricinus_communis | Euphorbiaceae | Angiosperms | dicots | black |
| XM002533940_Ricinus_communis | Euphorbiaceae | Angiosperms | dicots | black |
| CsMYBML64_Coronilla_scorpioides | Fabaceae | Angiosperms | dicots | blue |
| EV261021_Medicago_trunculata | Fabaceae | Angiosperms | dicots | blue |
| FG532960_Pisum_sativum | Fabaceae | Angiosperms | dicots | blue |
| FS274986_Glycyrrhiza_uralensis | Fabaceae | Angiosperms | dicots | blue |
| Glyma02g12260_Glycine_max | Fabaceae | Angiosperms | dicots | blue |
| Glyma13g05550_Glycine_max | Fabaceae | Angiosperms | dicots | blue |
| Glyma20g04240_Glycine_max | Fabaceae | Angiosperms | dicots | blue |
| Glyma7g35560_Glycine_max | Fabaceae | Angiosperms | dicots | blue |
| Glyma9g37040_Glycine_max | Fabaceae | Angiosperms | dicots | blue |
| LcMYBML64_Lotus_corniculatus | Fabaceae | Angiosperms | dicots | blue |
| Medtr4g112060_Medicago_trunculata | Fabaceae | Angiosperms | dicots | blue |
| Medtr6g013190_Medicago_trunculata | Fabaceae | Angiosperms | dicots | blue |
| RpMYBML64_Robinia_pseudoacacia | Fabaceae | Angiosperms | dicots | blue |
| VcMYBML64_Vicia_unijuga | Fabaceae | Angiosperms | dicots | blue |
| FP026752_Quercus_robur | Fagaceae | Angiosperms | dicots | black |
| CO090668_Gossypium_raimondii | Malvaceae | Angiosperms | dicots | purple |
| ES441147_Theobroma_cacao | Malvaceae | Angiosperms | dicots | purple |
| EU826465_Gossypium_hirsutum | Malvaceae | Angiosperms | dicots | purple |
| GhMML3_Gossypium_hirsutum | Malvaceae | Angiosperms | dicots | purple |
| GhMML7_Gossypium_hirsutum | Malvaceae | Angiosperms | dicots | purple |
| GhMYBML10_Gossypium_hirsutum | Malvaceae | Angiosperms | dicots | purple |
| GhMYBML4_Gossypium_hirsutum | Malvaceae | Angiosperms | dicots | purple |
| GrMYBML10_Gossypium_raimondii | Malvaceae | Angiosperms | dicots | purple |
| GrMYBML2_Gossypium_raimondii | Malvaceae | Angiosperms | dicots | purple |
| GrMYBML3_Gossypium_raimondii | Malvaceae | Angiosperms | dicots | purple |
| GrMYBML4_Gossypium_raimondii | Malvaceae | Angiosperms | dicots | purple |
| GrMYBML5_Gossypium_raimondii | Malvaceae | Angiosperms | dicots | purple |
| GrMYBML6_Gossypium_raimondii | Malvaceae | Angiosperms | dicots | purple |
| GrMYBML7_Gossypium_raimondii | Malvaceae | Angiosperms | dicots | purple |
| GrMYBML8_Gossypium_raimondii | Malvaceae | Angiosperms | dicots | purple |
| GrMYBML9_Gossypium_raimondii | Malvaceae | Angiosperms | dicots | purple |
| HM134083_Gossypium_raimondii | Malvaceae | Angiosperms | dicots | purple |
| XP_007032600.2_Theobroma_cacao | Malvaceae | Angiosperms | dicots | purple |
| XP_007046021.2_Theobroma_cacao | Malvaceae | Angiosperms | dicots | purple |
| XP_007046022.2_Theobroma_cacao | Malvaceae | Angiosperms | dicots | purple |
| XP_007046025.2_Theobroma cacao | Malvaceae | Angiosperms | dicots | purple |
| XP_017970561.1_Theobroma_cacao | Malvaceae | Angiosperms | dicots | purple |
| XP_017980167.1_Theobroma_cacao | Malvaceae | Angiosperms | dicots | purple |
| ES595589_Eucalyptus_globulus | Myrtaceae | Angiosperms | dicots | black |
| GO243958_Olea_europaea | Oleaceae | Angiosperms | dicots | black |
| GO959262_Mimulus_nasutus | Phrymaceae | Angiosperms | dicots | black |
| GO999094_Mimulus_nasutus | Phrymaceae | Angiosperms | dicots | black |
| MlMYBML7_Mimulus_lewisii | Phrymaceae | Angiosperms | dicots | black |
| DR923451_Aquilegia_formosa_x_pubescens | Ranunculaceae | Angiosperms | dicots | orange |
| DT728930_Aquilegia_formosa_x_pubescens | Ranunculaceae | Angiosperms | dicots | orange |
| FJ487606_Thalictrum_thalictroides | Ranunculaceae | Angiosperms | dicots | orange |
| GQ324997_Thalictrum_dioicum | Ranunculaceae | Angiosperms | dicots | orange |
| GQ324998_Thalictrum_filamentosum | Ranunculaceae | Angiosperms | dicots | orange |
| TtMYBML2_Thalictrum_thalictroides | Ranunculaceae | Angiosperms | dicots | orange |
| v1.006899m_Aquilegia_coerulea | Ranunculaceae | Angiosperms | dicots | orange |
| EB142336_Malus_domestica | Rosaceae | Angiosperms | dicots | black |
| EX658114_Fragaria_vesca | Rosaceae | Angiosperms | dicots | black |
| ppa006769m_Prunus_perica | Rosaceae | Angiosperms | dicots | black |
| DY296793_Citrus_clementina | Rutaceae | Angiosperms | dicots | black |
| POPTR_0008s08870_Populus_trichocarpa | Salicaceae | Angiosperms | dicots | black |
| POPTR_0010s17300_Populus_trichocarpa | Salicaceae | Angiosperms | dicots | black |
| AM833295_Nicotiana_tabacum | Solanaceae | Angiosperms | dicots | yellow |
| BF054497_Solanum_tuberosum | Solanaceae | Angiosperms | dicots | yellow |
| CA516461_Capsicum_anuum | Solanaceae | Angiosperms | dicots | yellow |
| FG639896_Nicotiana_tabacum | Solanaceae | Angiosperms | dicots | yellow |
| FS417072_Nicotiana_tabacum | Solanaceae | Angiosperms | dicots | yellow |
| GO605614_Nicotiana_benthamiana | Solanaceae | Angiosperms | dicots | yellow |
| PhMYB1_Petunia_hybrida | Solanaceae | Angiosperms | dicots | yellow |
| ScoMYBML2_Solanum_coriaceum | Solanaceae | Angiosperms | dicots | yellow |
| SelMYBML2_Solanum_elaeagnifolium | Solanaceae | Angiosperms | dicots | yellow |
| SsiMYBML2_Solanum_sisymbriifolium | Solanaceae | Angiosperms | dicots | yellow |
| StrMYBML2_Solanum_tridynamum | Solanaceae | Angiosperms | dicots | yellow |
| Z13996_Petunia_hybrida | Solanaceae | Angiosperms | dicots | yellow |
| XM002283807_Vitis_shuttleworthii | Vitaceae | Angiosperms | dicots | black |
| BY888796_Cryptomeria_japonica | Cupressaceae | Gymnosperms |  | black |
| EF087263_Picea_sitchensis | Pinaceae | Gymnosperms |  | black |
