# Supplementary figures and images for: Functional characterization of AarMIXTAs as essential regulators in T-shaped non-glandular trichome development of Artemisia argyi
Source: Adv Biotechnol (Singap). 2025 Sep 12;3(3):26. doi: 10.1007/s44307-025-00077-5 (PMC12425882; doi:10.1007/s44307-025-00077-5)

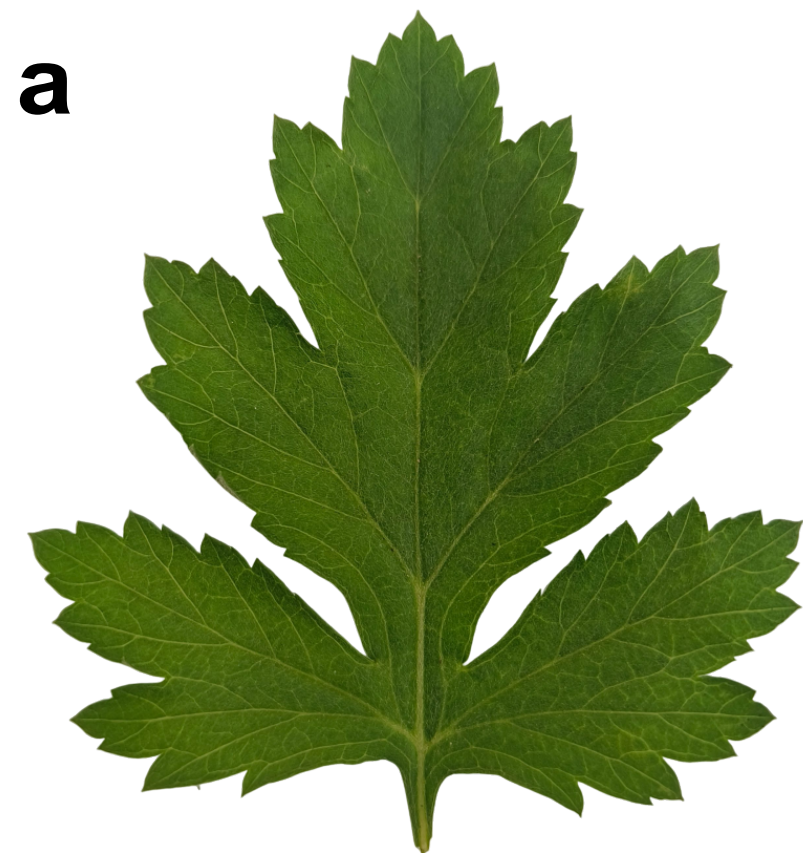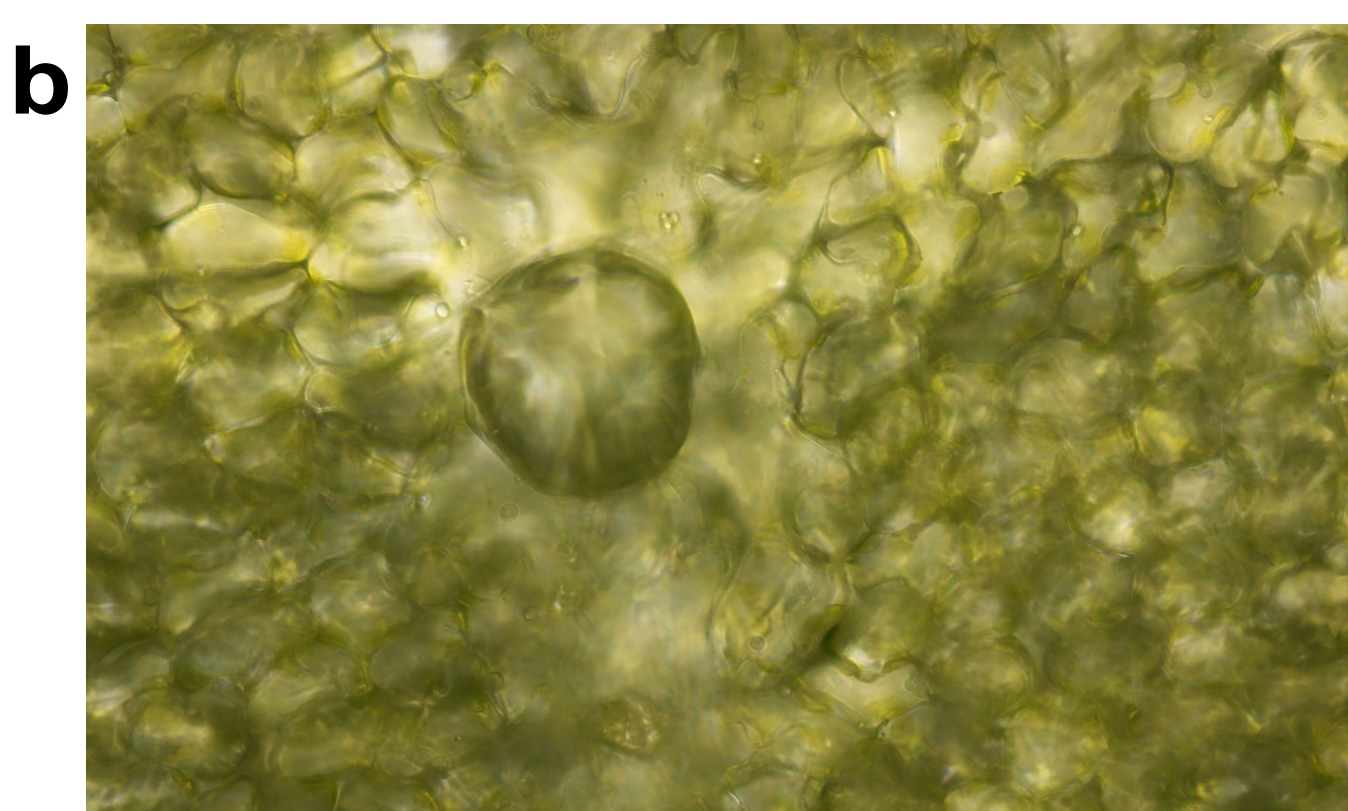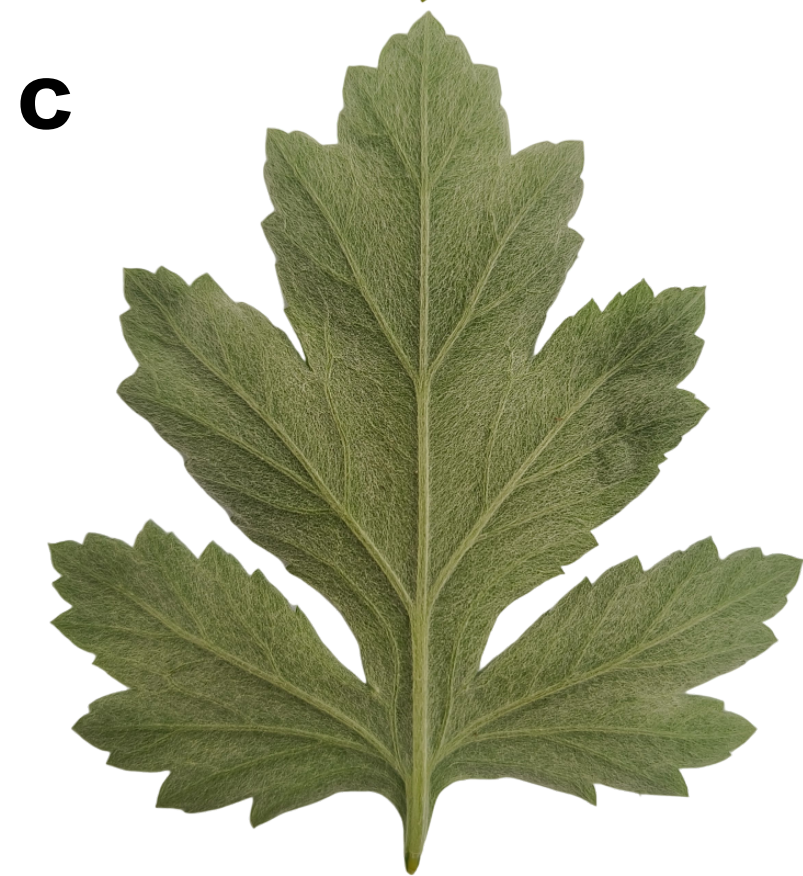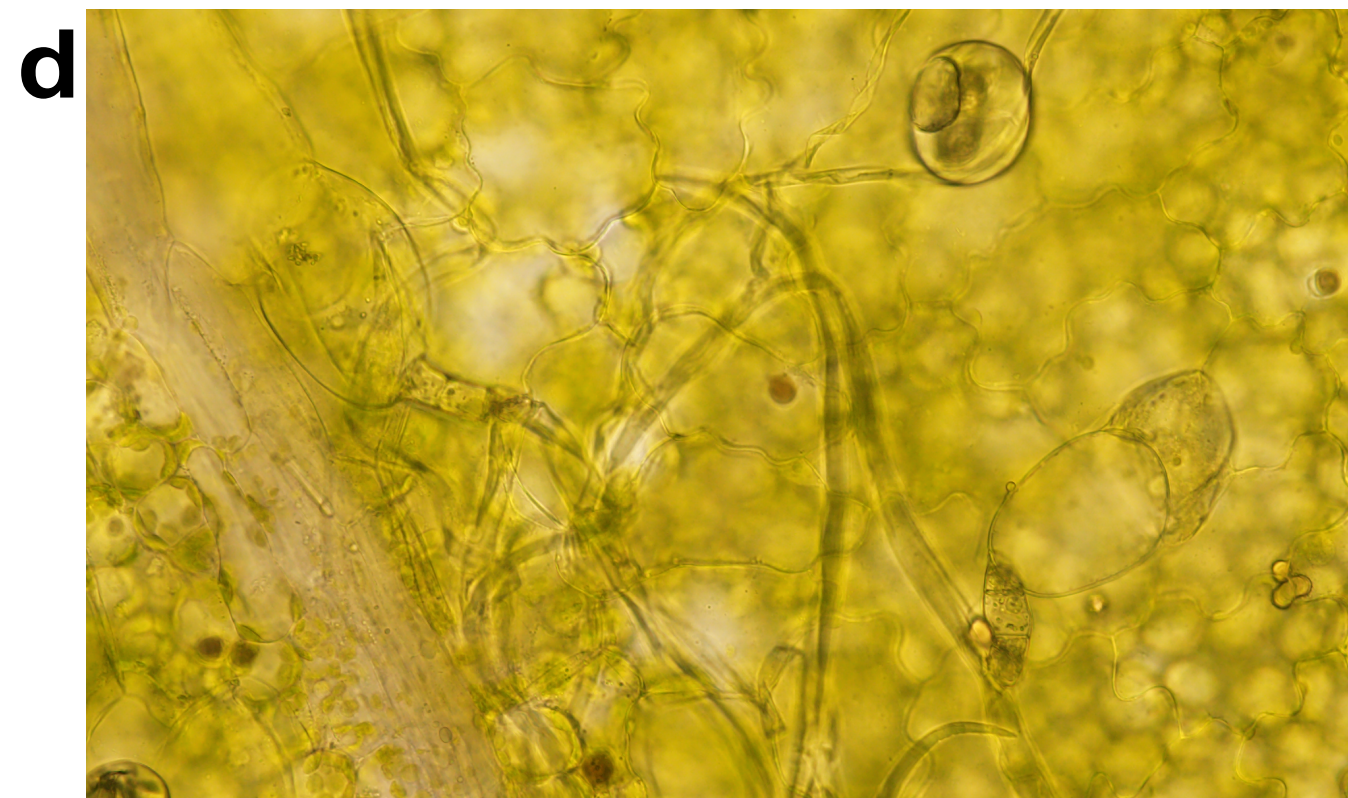

Supplement: Supplementary file 2 — Supplementary Material 2. FigS. 1 Microscopic observation of the epidermis of A. argyi. a Upper epidermis. b upper epidermis (Bright, fluorescence microscope ×40). c Lower epidermis. d Lower epidermis (Bright, fluorescence microscope ×40). [file 44307_2025_77_MOESM2_ESM.pdf]

**a**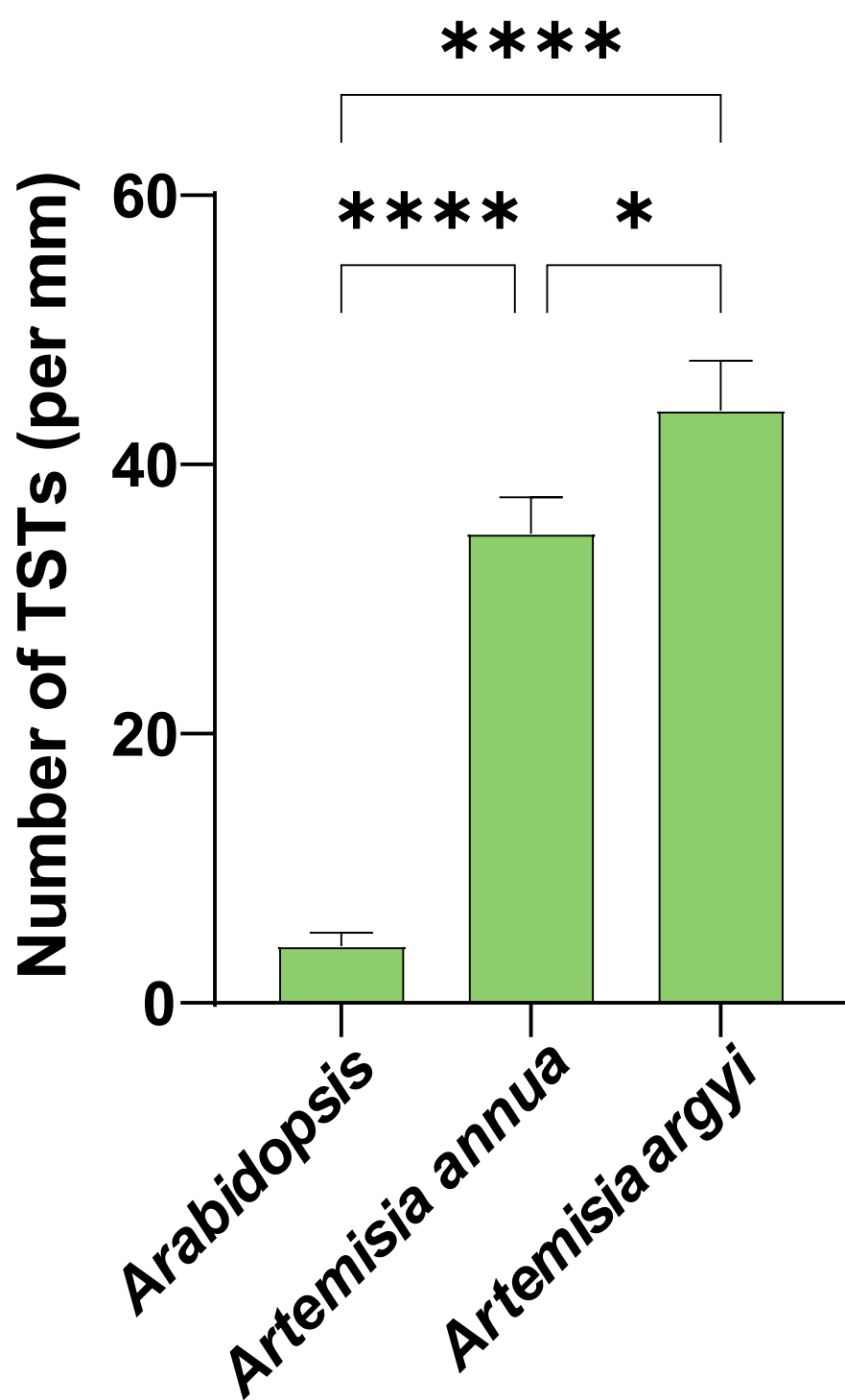**b**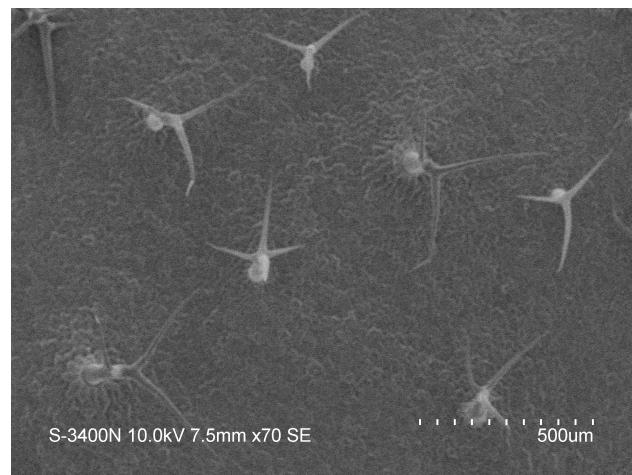**c**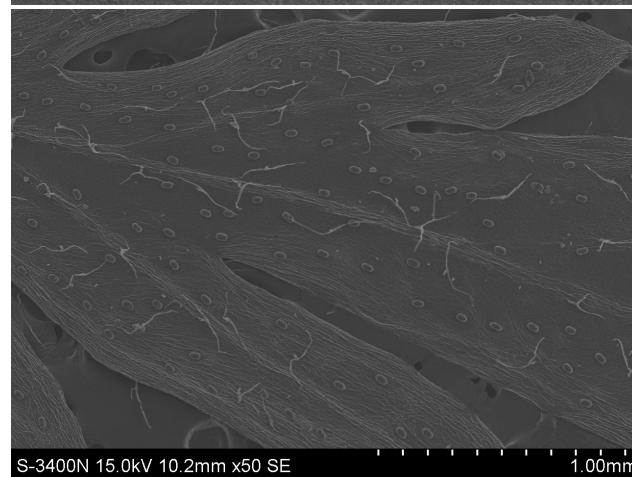**d**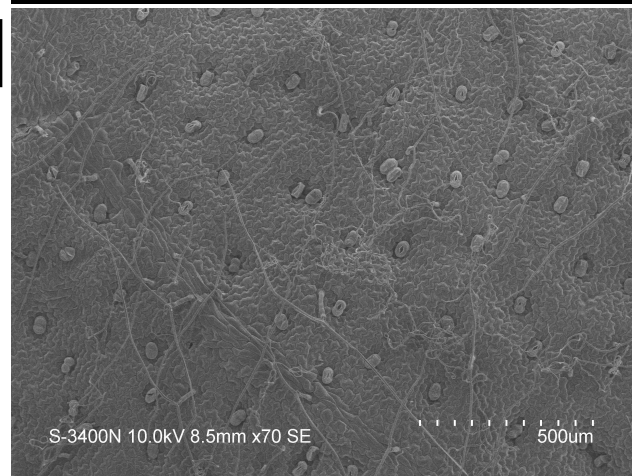

Supplement: Supplementary file 3 — Supplementary Material 3. FigS. 2 The density of TSTs on upper epidermis (a) and characteristics in (b) Arabidopsis, (c) A. annua and (d) A. argyi. [file 44307_2025_77_MOESM3_ESM.pdf]

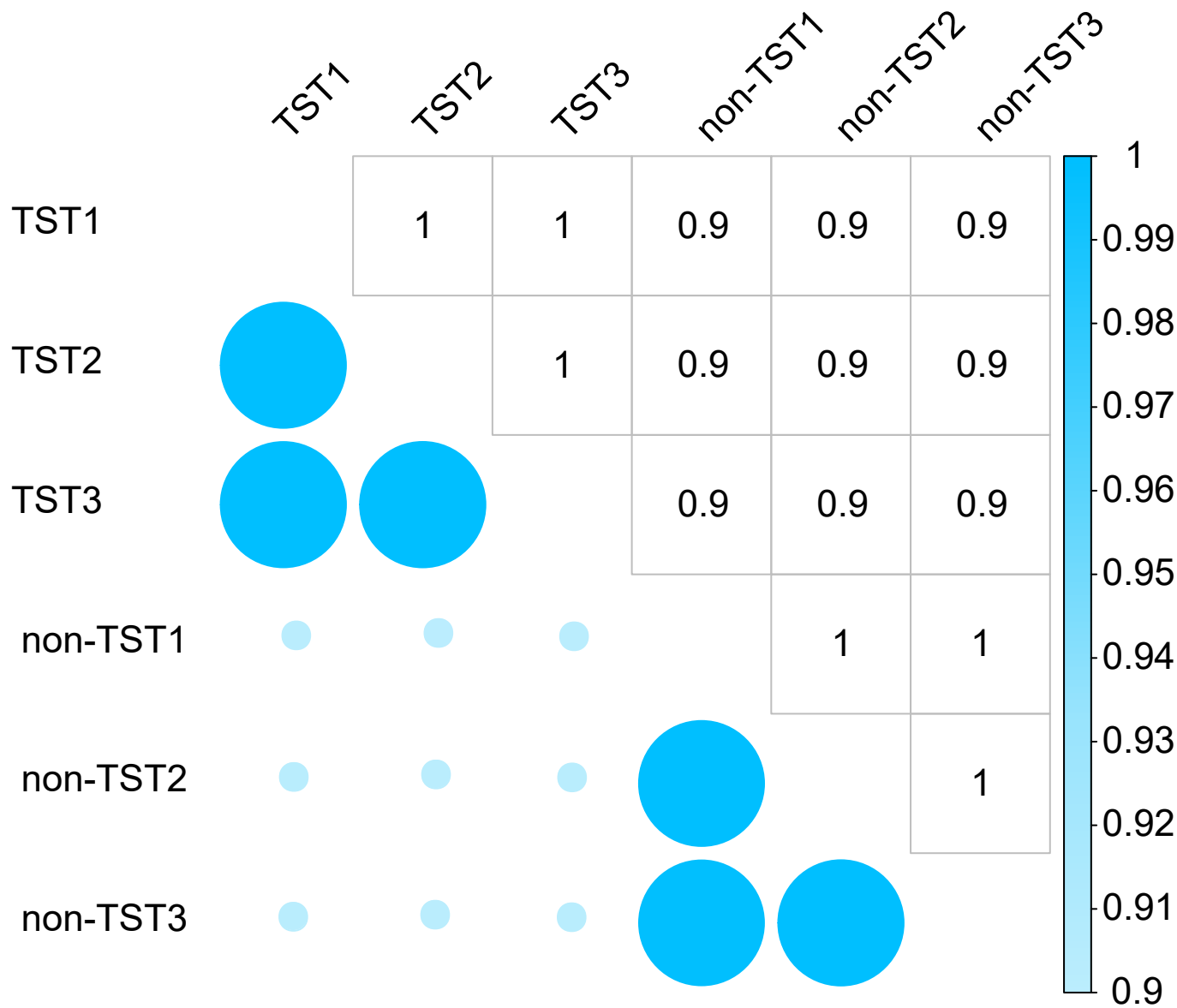

Supplement: Supplementary file 4 — Supplementary Material 4. FigS. 3 The correlation of sequenced samples. [file 44307_2025_77_MOESM4_ESM.pdf]

# Sample clustering to detect outliers

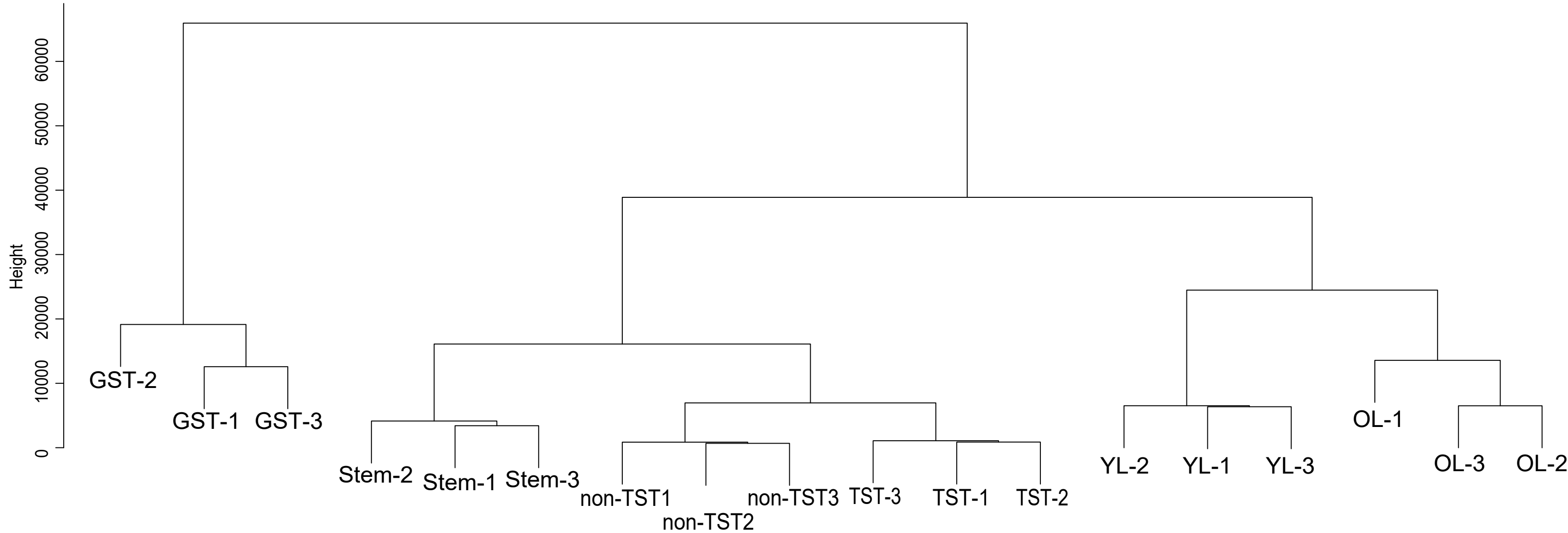

Supplement: Supplementary file 5 — Supplementary Material 5. FigS. 4 Sample clustering. GST: Trichome (NCBI); St: Stem; non-TST: mesophyll; TST: Trichome (ours); YL: Young_leave; OL: Old_leave. [file 44307_2025_77_MOESM5_ESM.pdf]

**M**

**-**

**35S::AarMIXTA1.2**

**2000**

**1000**

**750**

**500**

**200**

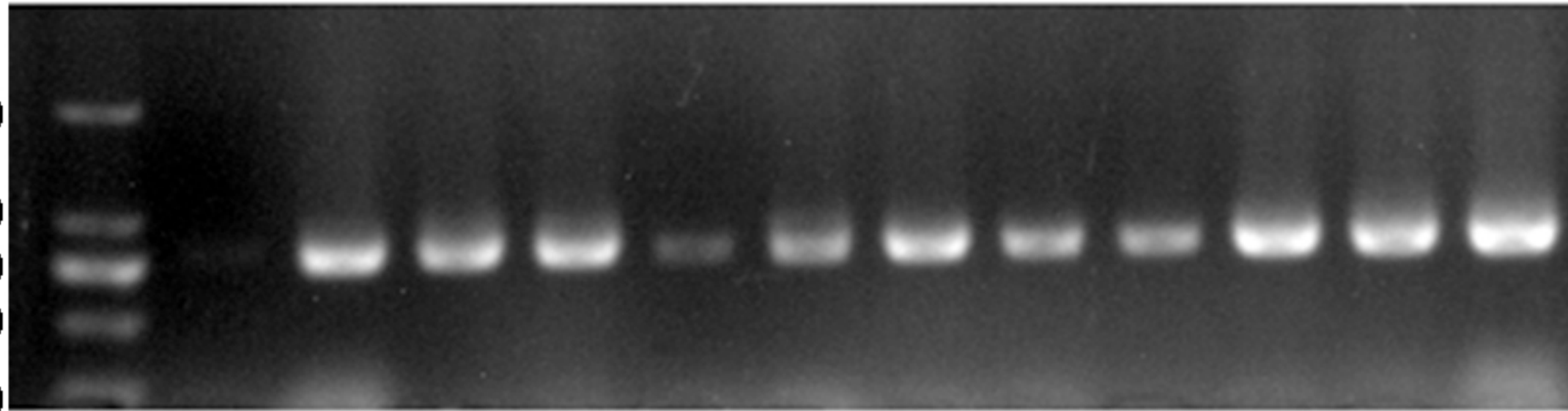

Supplement: Supplementary file 7 — Supplementary Material 7. FigS. 6 PCR verification of transgenic 35S::AarMIXTA1.2Arabidopsis. [file 44307_2025_77_MOESM7_ESM.pdf]
