# Supplementary material for: Functional characterization of AarMIXTAs as essential regulators in T-shaped non-glandular trichome development of Artemisia argyi
Source: Adv Biotechnol (Singap). 2025 Sep 12;3(3):26. doi: 10.1007/s44307-025-00077-5 (PMC12425882; doi:10.1007/s44307-025-00077-5)
Supplement: Supplementary file 6 — Supplementary Material 6. FigS. 5 Sequence similarity comparison of MIXTAs in Arabidopsis, A. annua and A. argyi. [file 44307_2025_77_MOESM6_ESM.pdf]

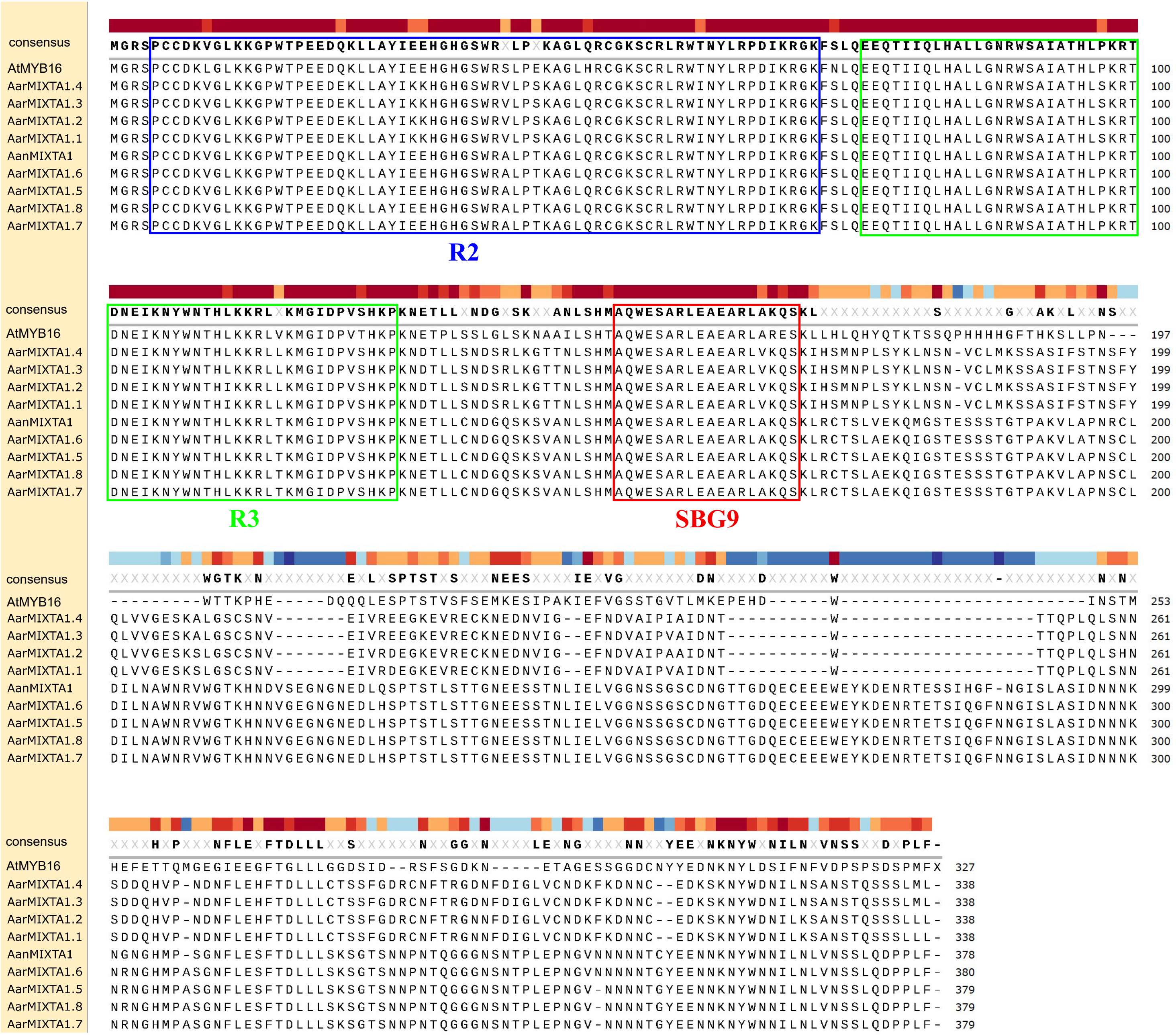
consensus
AtMYB16
AarMIXTA1.4
AarMIXTA1.3
AarMIXTA1.2
AarMIXTA1.1
AanMIXTA1
AarMIXTA1.6
AarMIXTA1.5
AarMIXTA1.8
AarMIXTA1.7
R2
R3
SBG9
